# mSphere of Influence: Adapting new models for studying *Toxoplasma gondii* gut interactions

**DOI:** 10.1128/msphere.00211-25

**Published:** 2025-06-06

**Authors:** Carlos J. Ramírez-Flores

**Affiliations:** 1 Department of Infectomics and Molecular Pathogenesis, Center for Research and Advanced Studies of the National Polytechnic Institute (Cinvestav), Mexico City, Mexico; Virginia-Maryland College of Veterinary Medicine, Blacksburg, Virginia, USA

**Keywords:** *Toxoplasma gondii*, intestinal colonization, intracellular parasites, 3D model, microfluidics, host-parasite interaction

## Abstract

Carlos J. Ramírez-Flores works in the field of parasitology, focusing on how *Toxoplasma gondii* crosses host barriers. In this mSphere of Influence article, he reflects on how three papers—by Shim, Kim, and Gregg—built on Dubey’s foundational mouse work to reshape his view of the parasite’s earliest intestinal events and to inspire his use of gut-on-chip platforms.

## COMMENTARY

### From plaques to chips

Nearly 30 years ago, J. P. Dubey showed that orally ingested *Toxoplasma gondii* oocysts invade the mouse small intestine, breaching enterocytes within hours and spreading from discrete foci to systemic infection (1). That classic work crystallized key questions that still vex us. How does the parasite traverse the intestinal barrier? Which host cues trigger its conversion from bradyzoites to tachyzoites, and how early does immune evasion begin? While animal studies map gross pathology, they struggle to isolate micro-domains of infection or to capture real-time dynamics at the epithelium–lamina propria interface. Ethical limits on human sampling compound the problem. Three papers published between 2013 and 2023 convinced me that new microphysiological and imaging approaches could finally illuminate this black box (2–4).

### What they discovered

Gregg et al. (2) revived Dubey’s oral infection model and, using multiphoton microscopy and flow cytometry, charted parasite loads along the duodenum, jejunum, and ileum during the first week (2). They revealed “multi-villus plaques”—expanding infection zones that foreshadow systemic spread and link tissue geography to disease outcome. Four years later, Shim et al. (3) engineered a microfluidic gut-on-a-chip whose collagen scaffolds protrude into the channel as three-dimensional villi (3). Although developed for drug-absorption studies, the device reproduced shear stress, brush-border maturation, and tight-junction integrity so faithfully that it immediately suggested a *Toxoplasma*-ready stage: a place where parasites could encounter villus tips under near-physiological flow. Kim et al. (4) seized that opportunity, stacking endothelial layers in a tri-channel chip and capturing tachyzoites as they pierced the endothelial barrier (4). Taken together, these models—traditional histology, 3D villus chips, and real-time transmigration assays—underline a single principle: micro-scale conditions set macro-scale disease. Early events at the intestinal interface, captured either as electrical drops *in vitro* or inflammatory plaques *in vivo*, decide how far and fast the parasite spreads.

### How they changed me

Gregg’s meticulous sectioning showed me that spatial mapping can still reveal surprises in a classic model; the notion of infection “plaques” became my mental template for early *Toxoplasma* biology. At the same time, I felt an urgent need for a system that could expose events impossible—or at least extremely difficult—to visualize in mice. Shim’s villus chip proved that organ-level geometry and physiological shear can be recreated *ex vivo*, convincing me that micro-engineering would let us witness the first dialog between parasite and epithelium without opening a mouse. Kim’s barrier-crossing device turned that promise into reality: it transformed the once-static concept of transmigration into measurable kinetics and demonstrated that on-chip analysis of *Toxoplasma* infection is feasible. These papers pulled me from a purely biological mindset into a multidisciplinary arena where biofabrication, live imaging, and parasitology intersect.

### Where do we go next

Human gut-on-chip platforms allow for the incorporation of oxygen gradients, commensal microbiota, and a variety of human-derived cells (5–7), while *in vivo* plaque mapping is now paired with single-cell transcriptomics (8). Fueled by these advances, my laboratory is adapting 3D models and villus-mimetic scaffolds to follow bradyzoite and tachyzoite dynamics in real time and to test how *Toxoplasma* manipulates its host to modulate early dissemination.
